# MiR-135a biogenesis and regulation in malignancy: a new hope for cancer research and therapy

**DOI:** 10.20892/j.issn.2095-3941.2020.0033

**Published:** 2020-08-15

**Authors:** Zhe Cao, Jiangdong Qiu, Gang Yang, Yueze Liu, Wenhao Luo, Lei You, Lianfang Zheng, Taiping Zhang

**Affiliations:** ^1^Department of General Surgery; ^2^Department of Nuclear Medicine, Peking Union Medical College Hospital, Chinese Academy of Medical Sciences and Peking Union Medical College, Beijing 100730, China; ^3^Clinical Immunology Center, Chinese Academy of Medical Sciences, Beijing 100730, China

**Keywords:** Biomarker, cancer, chemoresistance, epithelial-mesenchymal transition, miR-135a, therapeutics

## Abstract

MicroRNAs (miRNAs) are evolutionarily conserved small non-coding RNAs that affect posttranscriptional regulation by binding to the 3′-untranslated region of target messenger RNAs. MiR-135a is a critical miRNA that regulates gene expression, and many studies have focused on its function in cancer research. MiR-135a is dysregulated in various cancers and regulates cancer cell proliferation and invasion *via* several signaling pathways, such as the MAPK and JAK2/STAT3 pathways. MiR-135a has also been found to promote or inhibit the epithelial-mesenchymal transition and chemoresistance in different cancers. Several studies have discovered the value of miR-135a as a novel biomarker for cancer diagnosis and prognosis. These studies have suggested the potential of therapeutically manipulating miR-135a to improve the outcome of cancer patients. Although these findings have demonstrated the role of miR-135a in cancer progression and clinical applications, a number of questions remain to be answered, such as the dual functional roles of miR-135a in cancer. In this review, we summarize the available studies regarding miR-135a and cancer, including background on the biogenesis and expression of miR-135a in cancer and relevant signaling pathways involved in miR-135a-mediated tumor progression. We also focus on the clinical application of miR-135a as a biomarker in diagnosis and as a therapeutic agent or target in cancer treatment, which will provide a greater level of insight into the translational value of miR-135a.

## Introduction

MicroRNAs (miRNAs) are a group of short non-coding RNAs approximately 22 nucleotides in length^1^. Since the first discovery of miRNAs in 1993^2^, miRNAs have been highlighted as critical regulators of gene expression that act by binding to complementary target messenger RNAs (mRNAs)^3^. Mature miRNAs are incorporated into the RNA-induced silencing complex (RISC) after miRNA duplexes are processed by the proteins of the Argonaute (AGO) family^4,5^. The combination of mature miRNAs with the RISC enables miRNAs to target mRNAs with complementary sequences, leading to translational inhibition or mRNA degradation at the posttranscriptional level^6^. Accumulating studies have shown that miRNAs are involved in almost all physiological or pathological processes, including cellular proliferation and tumorigenesis^7,8^. MiR-135a is a member of the miR-135 family (which includes miR-135a and miR-135b) and is dysregulated in various cancers. MiRNAs can act as either oncogenes or tumor suppressors^9^, and recent studies have shown that miR-135a plays important but contradictory roles in cancer progression. MiR-135a acts as a promoter or inhibitor of cancer cell proliferation and invasion by regulating specific signal pathways and mRNAs. MiR-135a also modulates the epithelial-mesenchymal transition (EMT) and chemoresistance of cancer cells, which influences the outcomes of cancer patients. Therefore, miR-135a may be a promising molecule in cancer diagnosis and treatment. In this review, we focus on recent advances in studies of miR-135a effects on tumor progression and its potential applications in clinical practice as a novel approach in cancer therapy.

## Biogenesis and expression of miR-135a in cancer

**Figure 1:**
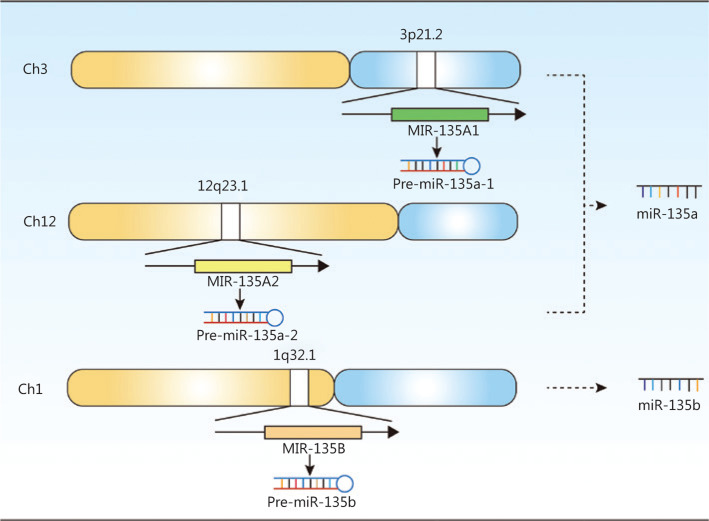
The gene expression of the miR-135 family in humans. Two genes, *MIR-135A1* and *MIR-135A2*, were located at different chromosomes in humans, and they could be transcribed and translated into miR-135a. Only one gene, *MIR-135B*, was responsible for the expression of miR-135b in humans.

The miR-135 family includes miR-135a and miR-135b. MiR-135a is encoded by the *MIR135A1* gene (Gene ID: 406925), located on human chromosome 3p21.2, and *MIR135A2* (Gene ID: 406926), located on human chromosome 12q23.1; miR-135b is encoded by the *MIR135B* gene (Gene ID: 442891), which is located on human chromosome 1q32.1. **Figure 1** shows the gene expressions of the miR-135 family.

Genes encoding miR-135a are transcribed into primary microRNAs (pri-miRNAs) *via* RNA polymerase II, and pri-miRNAs are then cleaved by Drosha, a ribonuclease III enzyme, and its cofactor DGCR8 to produce stem-loop precursor miRNAs (pre-miRNAs) that contain approximately 70 nucleotides^10^. Exportin-5 is then responsible for transporting the pre-miRNAs from the nucleus into the cytoplasm, where the terminal loop of pre-miRNA is cleaved by the RNase III Dicer and TAR RNA binding protein, resulting in miRNA duplexes^10–12^. The miRNA duplexes are incorporated into RISC and processed by AGO protein into mature miRNAs^3^. Mature miR-135a can also be transported to the intercellular space or circulation to exert regulatory effects on nearby or remote cells. Mature miRNAs can be transported in a free form or by transportation mediators such as exosomes, which carry biological contents from donor to recipient cells and release biological contents *via* cell membrane fusion^13^. A previous study showed that circulating exosomal miR-135a served as a biomarker for disease diagnosis^14^.

MiRNA dysregulation has been reported in various diseases, especially in cancer^8,15^, and is possibly attributed to genetic and epigenetic alterations, dysregulated transcription factors, or mutations in genes encoding miRNA-biogenesis enzymes^16–18^. The aberrant expression of miR-135a in cancer was first reported in colorectal adenoma and adenocarcinoma tissues^19^. Compared with normal colon epithelium, colorectal adenomas showed a 3.2-fold increased expression of miR-135a (*P* < 0.001) and colorectal adenocarcinomas showed a 6.7-fold increase (*P* < 0.001)^19^. Similar results were obtained in both colorectal cancer cell lines and patient serum^20,21^. Upregulation of miR-135a was also found in ependymoma^22^, breast cancer^23^, bladder cancer^24^, melanoma^25^, and hepatocellular carcinoma^26^, indicating that miR-135a may serve as a tumor promoter in these cancers.

In contrast, other studies reported that miR-135a was downregulated in several cancer types. MiRNA profiling by miRNA arrays in prostate cancer, ovarian cancer, and pancreatic cancer showed a dramatic decrease in miR-135a expression in serum or cell lines compared to that in controls^27–29^. Lung cancer clinical specimens, serum, and cells all contained decreased levels of miR-135a^30–33^. MiR-135a was also significantly decreased in thyroid carcinoma^34^, renal cell carcinoma^35^, gall bladder cancer^36^, osteosarcoma^37^, and glioma^38^. In addition, decreased miR-135a was one of the miRNA changes that correlated with familial papillary thyroid carcinoma^39^.

Even within the same cancer type, the expression of miR-135a can differ dramatically. Although two independent studies using RT-PCR reported that miR-135a was decreased in both tissues and cell lines of gastric cancer^40,41^, Yan et al.^42^ found that miR-135a was upregulated in gastric cancer using 280 paired clinical samples. We speculate that the discrepancy regarding the expression of miR-135a may be attributed to the dramatic difference in sample volumes or patient states (with or without chemotherapy before surgery), but the underlying mechanisms remain to be further explored.

## Mechanisms of miR-135a dysregulation in cancer

The studies discussed above indicate that miR-135a expression varies among cancers, and therefore, exploring the mechanisms regulating miR-135a expression is fundamental to clarifying its functions in cancer. Current studies regarding the regulation of miR-135a expression have mainly focused on its transcription from coding genes, which is affected by several transcription factors. For example, androgen receptor (AR) activates the transcription of miR-135a by directly binding to androgen response elements (AREs) in the promoter region of the *MIR135A2* gene in prostate cancer cell lines; the ARE sequence, CAAGTACAGCTTGTTCTCC, is located -5,605 bp upstream of pre-miR-135a2^43^. Another study revealed that the transcription of miR-135a was activated by signal transducer and activator of transcription 5a (STAT5a), a member of the STAT family, which binds to both promoter elements of miR-135a-1 and miR-135a-2 genes in 3T3-L1 cell lines^44^. The binding sites for STAT5a are at -874/-856 and -2020/-2002 in the miR-135a-1 and miR-135a-2 genes, respectively^44^. A similar mechanism exists in the regulation of miR-135a expression by p53^45^.

Other studies have shown that miR-135a is also regulated by interactions with long non-coding RNAs (lncRNAs), such as urothelial carcinoma associated 1 (UCA1)^46^, gastric cancer associated transcript 3 (GACAT3)^47^, FOXD3 antisense RNA 1 (FOXD3-AS1)^48^, differentiation antagonizing nonprotein coding RNA (DANCR)^49^, muscleblind-like 1 antisense RNA 1 (MBNL1-AS1)^50^, and the small nucleolar RNA host gene 16 (SNHG16)^51^. These lncRNAs act as competing endogenous RNAs (ceRNAs) by binding to miRNAs *via* miRNA response elements^52^. Bioinformatics analysis revealed two complementary sites of miR-135a in UCA1, and a luciferase activity assay confirmed that miR-135a was a target of UCA1 and was decreased by UCA1^46^. Similarly, GACAT3 decreased miR-135a expression by acting as a ceRNA^47^, similar to FOXD3-AS1 and MBNL1-AS1^48,50^. MiR-135a is also sponged by SNHG16, and this process is promoted by activation of the JAK2/STAT3 pathway^51^. The inhibition of miR-135a by DANCR was also reported in pancreatic cancer and tongue squamous cell carcinoma cells, which resulted in promoted cancer cell proliferation and invasion^49,53^. MiR-135a was also predicted to interact with lncRNA KIAA0125 and may participate in the development of ameloblastoma^54,55^. The circular RNA-like Cdr1as was also shown to sponge and inhibit miR-135a activity by directly binding to miR-135a^56^.

Epigenetic alterations have emerged as another important cause of miR-135a dysregulation. Promoter hypermethylation of miR-135a was detected in microsatellite-unstable colorectal cancer cell lines and decreased the expression of miR-135a^57^. Increased methylation at or near miR-135a-associated CpG islands was also discovered in multiple myeloma, and the demethylating agent, 5-azacytidine, rescued the expression of miR-135a^58^.

Hepatitis B X-interacting protein (HBXIP) has been shown to upregulate miR-135a^59^, and the PI3K/AKT/mTOR signaling pathway has been shown to decrease miR-135a expression^60^, but the exact mechanisms remain unknown.

**Figure 2:**
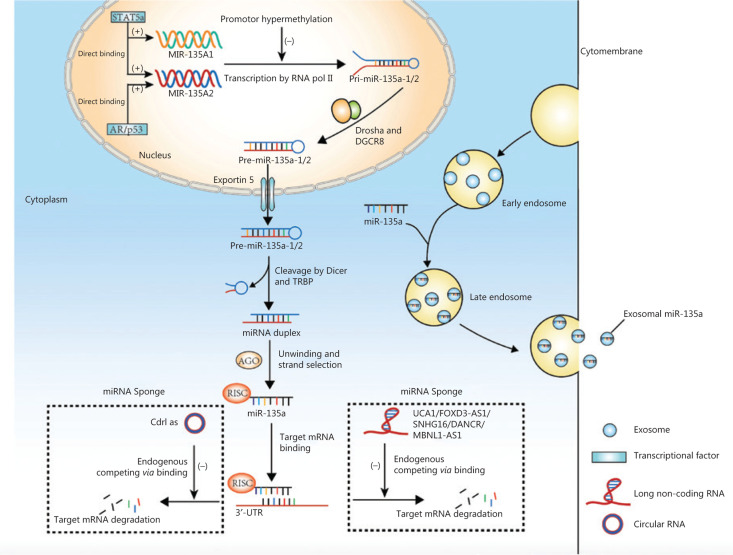
The mechanisms of biogenesis and dysregulation of miR-135a in cancer. The gene encoding miR-135a was transcribed into primary microRNAs (pri-miRNA), which was affected by transcription factors and promoter hypermethylation. The pri-miRNA was processed into precursor miRNAs by Drosha and DGCR8. The mature miRNA could be sponged by circular RNA and long non-coding RNA, to inhibit the degradation of target mRNA. The mature miRNA could also be transported into the circulation by exosomes.

Above all, the regulation of miR-135a expression involves transcription, interactions with lncRNAs. and epigenetic alterations. **Figure 2** summarizes the mechanisms of biogenesis and dysregulation of miR-135a in cancer.

## Biological function of miR-135a in cancer and the relevant signaling pathways

Studies on the biological function of miR-135a in cancer have shown conflicting results. The varying dysregulations of miR-135a among different cancers indicate that miR-135a has both oncogenic and anti-oncogenic effects, which have been verified by studies on cellular proliferation, tumor progression, and chemoresistance. Here, we discuss the dual role of miR-135a in cancer and its downstream targets, and summarize the relevant signaling pathways involved in this process.

### The miR-135a modulates cell proliferation and cancer progression

MiR-135a plays different roles in cellular proliferation and cancer progression, depending on the cancer type. Thus far, most studies have indicated that miR-135a has positive effects on cellular proliferation and cancer progression, while a few studies have reported opposite conclusions.

The antitumor effects of miR-135a are mediated by its targeting of various oncogenes, and the associated pathways include the p38 MAPK and JAK2/STAT3 pathways. MiR-135a causes G0/G1 arrest and inhibits the proliferation and invasion of thyroid carcinoma cells by binding to the 3′-untranslated region (3′-UTR) of Versican, and *in vivo* experiments revealed that miR-135a decreased the size of tumors^34^. A similar inhibitory effect of miR-135a was revealed in pancreatic cancer by its targeting of Bmi-1^28^ and in gallbladder cancer by its targeting of very low density lipoprotein receptor (VLDLR); the p38 MAPK pathway is the downstream signaling pathway of miR-135a-VLDLR^36^. MiR-135a suppressed cell proliferation and tumor growth in an ovarian cancer mouse xenograft model^27^, and this effect may be attributed to the regulation of C-C chemokine receptor type 2 (CCR2) by miR-135a^61^. The role of miR-135a in urological cancers has been intensively investigated. In renal cell carcinoma, miR-135a reduced cell viability by targeting C-Myc and JAK2, thus blocking the JAK2/STAT3 pathway^35,62^. A similar mechanism was discovered in classic Hodgkin’s lymphoma, in which JAK2 was decreased by miR-135a, leading to downregulation of the Bcl-xl anti-apoptotic gene^63^. In prostate cancer, miR-135a repressed cell migration and proliferation by downregulating matrix metallopeptidase 11 (MMP11), rho associated coiled-coil containing protein kinase 1 (ROCK1), ROCK2, RB associated KRAB zinc finger (RBAK) and STAT6^43,60,64^. In metastatic prostate cancer, miR-135a inhibited tumor progression by decreasing epidermal growth factor receptor (EGFR)^29^, and its silencing resulted in increased AR axis activity, thus contributing to disease progression^65^. In the genesis of glioma, miR-135a caused G1 arrest and reduced cell proliferation by targeting tumor necrosis factor receptor-associated factor 5 (TRAF5) and subsequently blocking AKT phosphorylation and the expression of c-Myc and cyclin D1^66^. In addition, the migratory capacity of glioblastoma cells was decreased by miR-135a mediated by downregulation of the Na^+^/H^+^ exchanger isoform 9 (NHE9), which acidified the endosomes that transport EGFR to the cell membrane, thus decreasing the activity of EGFR^67^. The antitumor effect of miR-135a in breast cancer was shown by its targeting of estrogen-related receptor α (ERRα), ETS transcription factor 1 (ELK1), and ELK3^68,69^. Other targets involved in miR-135a-induced tumor suppression include IL-17 in nasopharyngeal carcinoma and homeobox A10 (HOXA10) in head and neck squamous cell carcinoma^70,71^.

However, in other cancers, miR-135a acts as a tumor promoter *via* the following mechanisms: upregulating oncogene expression, blocking cancer suppresser genes, and serving as the target of oncoproteins. In hepatocellular carcinoma, for example, miR-135a increases the expression of oncogenes and the phosphorylation of AKT to promote the migration and invasion of HCC cells^26^, but it suppresses the forkhead box O1 (FOXO1) tumor suppressor, which results in a decrease in phosphoenolpyruvate carboxykinase 1 (PCK1), a key enzyme in gluconeogenesis^26,59^. A similar relationship between miR-135a and FOXO1 was discovered in malignant melanoma^72^. MiR-135a is also increased by oncoprotein hepatitis B X-interacting protein (HBXIP) and forkhead box M1, thus blocking the expression of the metastasis suppressor 1 (MTSS1)^59,73^. MiR-135a is involved in hepatitis C virus-mediated hepatocarcinogenesis with its decreasing protein tyrosine phosphatase receptor delta expression to promote the activity of STAT3^74^. Similarly, the inhibition of MTSS1 by miR-135a has also been observed in colorectal cancer^20^, and tumorigenesis may be partly attributed to the targeting of adenomatous polyposis coli (APC) by miR-135a, which induces the activation of the Wnt pathway^19^. In bladder cancer, miR-135a promotes cell proliferation by targeting pleckstrin homology domain leucine-rich repeat protein phosphatase (PHLPP2) and FOXO1, with downregulation of p21 and p27 and upregulation of cyclin D1^24^. In addition, it accelerates the migration and invasion of bladder cancer cells by downregulating glycogen synthase kinase 3 beta (GSK3β) to activate the Wnt/β-catenin pathway^75^. In cervical cancer, miR-135a induces malignant transformation in HPV-infected cells and the progression of tumors by targeting seven in absentia homologue 1 (SIAH1), contributing to the activation of the β-catenin/T cell factor signaling pathway^76^.

**Figure 3:**
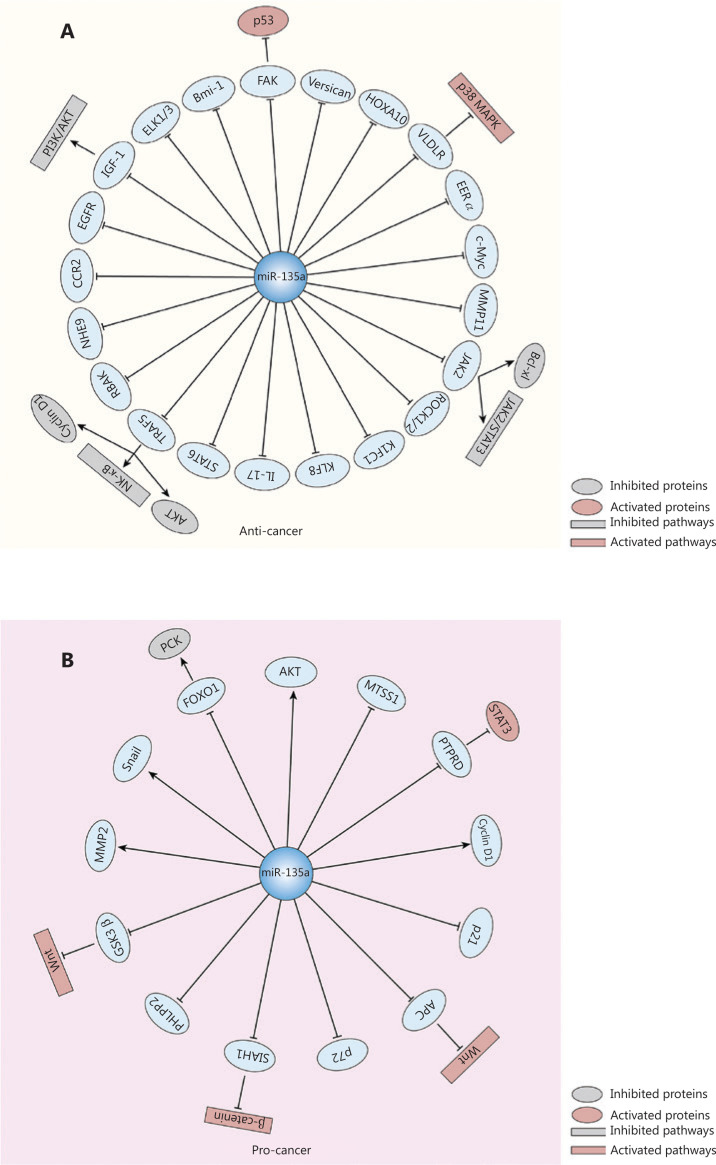
The effects of miR-135a on cancer progression and relevant targets and signaling pathways. (A) The miR-135a could inhibit the progression of cancer by targeting various mRNAs, which led to the inactivation of several pathways, including the PI3K/AKT pathway, JAK2/STAT3 pathway, and NF-κB pathway, as well as the activation of the p38 MAPK pathway and the p53 cancer suppressor gene. (B) The miR-135a can also promote cancer development *via* the activation of the Wnt/β-catenin pathway and STAT3, and the inactivation of phosphoenolpyruvate carboxykinase.

Several studies also reported that miR-135a exhibits both pro- and antitumor effects in some specific cancer types, such as gastric cancer and lung cancer. In gastric cancer, the proliferation of cancer cells is inhibited by miR-135a *via* inactivation of JAK2/STAT3 and targeting cyclin D1, Bcl-xl^40^, kinesin family member C1^41^ and the focal adhesion kinase (FAK) pathway^45^. In addition, the migration of gastric cancer can be inhibited by miR-135a *via* its targeting of TRAF5, which subsequently blocks the NF-κB pathway^77^. However, Yan et al.^42^ found that a high level of miR-135a was related to poor survival and shorter time to recurrence. It promotes oxaliplatin-resistant gastric cancer cell invasion and proliferation by decreasing E2F transcription factor 1 (E2F1) and death-associated protein kinase 2 (DAPK2) expression. In lung cancer, miR-135a induces apoptosis and inhibits invasion and angiogenesis by binding to the 3′-UTR of insulin-like growth factor 1 (IGF-1) mRNA and inactivating the IGF-1/PI3K/AKT signaling pathway^78^. It can also regulate EMT-related markers, such as E-cadherin and vimentin, by targeting Kruppel-like factor 8 (KLF8)^31^. However, several studies found that miR-135a contributed to lung cancer progression by targeting lysyl oxidase-like 4 (LOXL4)^79^ and promoting the chemoresistance of lung cancer^80,81^. **Figure 3** summarizes the effects of miR-135a on cancer progression.

**Figure 4:**
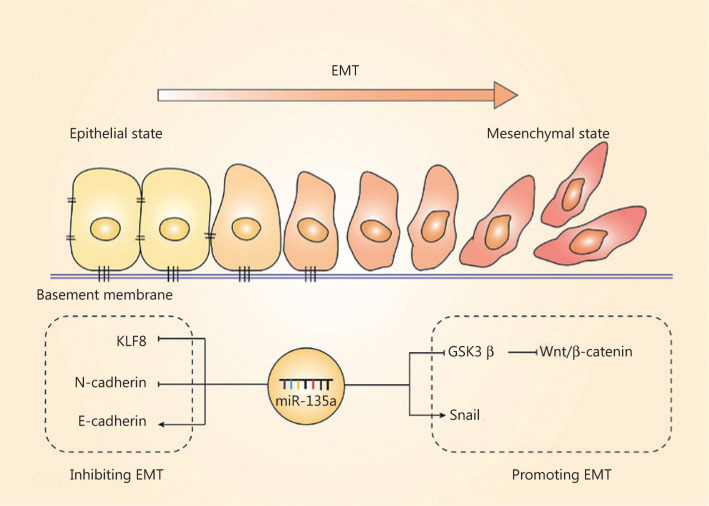
The effect of miR-135a on the epithelial-mesenchymal transition (EMT). The role of miR-135a on the EMT was contradictory. The miR-135a inhibited the EMT by decreasing KLF8 and N-cadherin, and increased the epithelial marker, E-cadherin. In contrast, miR-135a promoted the EMT *via* upregulating Snail and activating the Wnt/β-catenin pathway.

The EMT, another important hallmark of cancer cells, is the process by which cell morphology, biological behavior changes, and cancer cells acquire the ability for invasion and metastasis^82–84^; this process is also regulated by miR-135a. The EMT is characterized by the repression of epithelial genes, and loss of E-cadherin is a crucial step of the EMT^85^. The regulation of the EMT by miR-135a mainly relies on EMT-related transcriptional factors and pathways, and most of the present studies have indicated that miR-135a plays a suppressive role in the EMT. MiR-135a inhibits the EMT in non-small cell lung cancer (NSCLC) cells by directly binding to the 3′-UTR of KLF8^31^. Overexpression of miR-135a in ovarian cancer was also reported to inhibit EMT by reducing the level of N-cadherin and increasing the expression of E-cadherin, resulting in suppressed metastasis *in vivo*^61^. In contrast, miR-135a was found to accelerate the EMT and invasion in bladder cancer by targeting GSK3β and activating the Wnt/β-catenin pathway^75^. The relationship between the EMT and miR-135a remains to be further investigated, and these results should increase our understanding of the mechanisms of cancer progression. **Figure 4** summarizes the role of miR-135a in the EMT.

In summary, the present studies revealed the oncogenic and antitumor roles of miR-135a in different cancer types. Notably, conflicting roles and expression levels of miR-135a have also been observed, even in the same cancer type, as in gastric cancer. Considering that cancer cells from different organs may have completely different biological features and genetic contexts, it seems explainable by the unique role of miR-135a, because the expression levels of miR-135a target genes vary among these cancer cells. However, the conflicting roles of miR-135a in the same cancer type might be attributed to the different origins of cell types within the organ, and different clinical stages may also have an impact. The sample sizes of the present studies were relatively small, and further studies are therefore needed to evaluate the effects of miR-135a on cancer because the conflicting roles will present a challenge for the design of therapies that target miR-135a.

### The miR-135a and chemosensitivity

Chemotherapy is currently an essential treatment for cancer, and includes neoadjuvant chemotherapy and adjuvant chemotherapy. However, chemoresistance remains the main obstacle limiting the therapeutic effect of chemotherapy, especially in pancreatic cancer and ovarian cancer^13,86–88^. The process of chemoresistance includes several biological processes, such as cellular apoptosis, cell cycle progression, the EMT, and drug efflux, involving changes in multiple genes^89^. The mechanisms underlying chemoresistance have been intensively investigated, and miRNAs have been found to play an important role *via* the regulation of relevant genes^90,91^. MiR-135a has both anti- and pro-chemoresistance effects in cancer, depending on the specific cancer cell lines and target genes. *In vitro* studies reported that ovarian cancer cells transfected with the miR-135a vector showed decreased viability when compared with controls transfected with blank vector, and the IC_50_ of paclitaxel was lower in ovarian cancer cells transfected with miR-135a, which may be explained by the direct regulation of protein phosphatase 2 regulatory subunit B beta expression and indirect regulation of baculoviral IAP repeat containing 3, gamma-aminobutyric acid receptor subunit α3 or sperm protein associated with the nucleus on the X chromosome B1/2 expressions by miR-135a^27^. In lung cancer, miR-135a was shown to increase the sensitivity of cancer cells to cisplatin by directly targeting MCL1 and reducing the expression of MCL1^32^. However, studies have also shown that miR-135a contributes to chemoresistance in cancer cell lines^42,81^. MiR-135a was upregulated in oxaliplatin-resistant gastric cancer samples and was related to advanced pathological stage, vascular invasion, lymphatic vessel metastasis, and early recurrence^42^. The overexpression of miR-135a enhanced resistance to oxaliplatin both *in vivo* and *in vitro* by inhibiting E2F transcription factor 1 (E2F1) expression and the Sp1/death associated protein kinase 2 (DAPK2) signaling pathway^42^. Overexpression of miR-135a was also observed in drug resistant NSCLC, breast cancer, and prostate cancer cell lines compared with parental cell lines^81^. The chemoresistance of these cancers was enhanced by miR-135a, which was in part attributed to reduced APC expression^81^.

Recent studies have found that miRNAs also act as predictors of chemosensitivity and targets for overcoming chemoresistance in the treatment of cancer^92–94^. In breast cancer, miR-135a was shown to be a potential indicator of drug sensitivity^95^. The delivery of miRNA mimics or inhibitors to restore the normal gene network may sensitize cancer cells to chemotherapy^93,94^. Therefore, chemotherapeutic drugs combined with miRNA mimics or antagomirs may be a novel strategy for cancer treatment. However, studies on miR-135a in chemoresistance are still at an early stage, and the application of miR-135a to inhibit chemoresistance still remains to be explored. Further studies are therefore needed.

## The clinical applications of miR-135a

The abnormal expressions of miR-135a in various cancers indicate that it may be a potential biomarker for cancer diagnosis. In addition, several studies have shown that miR-135a expression is related to tumor node metastasis (TNM) stage, recurrence, and survival. Based on the regulatory effects of miR-135a on cancer cell proliferation, apoptosis, and chemoresistance, miR-135a may also be a promising therapeutic target in cancer treatment.

### The miR-135a as a biomarker for cancer diagnosis and prognosis

The present methods for cancer screening, including serum tumor markers and ordinary imaging examinations, lack high sensitivity and specificity. The final diagnosis of cancer relies on pathology or cytology, which is invasive and costly. Compared with these methods, miRNAs have some advantages as biomarkers for cancer^96^. First, miRNAs are easily accessible in body fluids, such as serum and urine, providing a noninvasive and economical method for diagnosis^97^. Second, miRNAs show high stability and resistance to degradation in circulation, even in paraffin-embedded tissues^98^. In addition, different cancers have distinctive miRNA profiles that differentiate cancer from benign diseases^99^. As previously mentioned, distinguishable abnormalities in miR-135a expression have been identified in numerous cancer types, thus providing strong rationale for its application as a diagnostic or prognostic biomarker.

Previous studies showed that the levels of miR-135a in serum, tissue samples, and peritoneal fluid of ovarian cancer patients were decreased significantly compared with those in ovarian cysts and normal ovaries, indicating that miR-135a may be a biomarker for ovarian cancer diagnosis^27^. In the diagnosis of colorectal cancer, serum miR-135a has the ability to distinguish colorectal cancer from colorectal polyps, with an area under the curve (AUC) of 0.832, and from healthy tissue, with an AUC of 0.875^21^. The combination of miR-21, miR-135a, miR-335, miR-206, and let-7a detected the presence of metastasis with a specificity of 87% and sensitivity of 76%^100^. A miRNA signature including miR-135a in serum exosomes was also able to distinguish between very high risk prostate cancer and low risk prostate cancer or healthy individuals, with an accuracy of at least 89%^101^. The low expression of miR-135a could also discriminate between metastatic and primary prostate cancers, with an AUC of 0.877 and was related to a high Gleason score^29^. Aside from serum miRNAs, a urinary miRNA signature including miR-135a was shown to accurately predict bladder cancer^102^. In the training set of the study, 14 miRNAs were selected as potential biomarkers from 16 urine samples (including four low grade bladder cancers, four high grade bladder cancers, and eight normal controls), but the validation set including 202 urine samples (115 bladder cancers and 87 normal controls) identified a subset of six urinary miRNAs (downregulated: let-7c, miR-148a, and miR-204; upregulated: miR-135a, miR-135b, and miR-345) that correctly differentiated bladder cancer from normal controls with an AUC of 0.883. The miRNA signature also distinguished between different subgroups and normal controls^102^. **Table 1** summarizes the studies on miR-135a as a biomarker for cancer diagnosis.

In addition to its value in cancer diagnosis, miR-135a has potential application in predicting the prognosis. Most of the current studies have indicated that miR-135a expression is positively correlated with the prognosis. For example, ovarian cancer patients with high serum miR-135a levels had a favorable prognosis, and low levels of miR-135a were correlated with poor survival and progression-free survival in ovarian cancer^27,103^. Decreased expression of miR-135a was also associated with poor survival in gastric cancer^45^. Nevertheless, another study found that higher expression of miR-135a in tumor tissues of gastric cancer was correlated with shorter survival and shorter time to recurrence^42^. In NSCLC, the serum miR-135a level was closely related to distant metastasis, lymphatic metastasis, TNM stage, and pathological stage. Patients with low miR-135a levels had significantly shorter survival than those with high miR-135a levels, and miR-135a was an independent risk factor for NSCLC prognosis^30^. In classic Hodgkin lymphoma, patients with low miR-135a expression had a higher incidence of recurrence and a shorter disease-free survival^63^. In hepatocellular carcinoma, however, high tumor expression of miR-135a was correlated with a high risk of recurrence within 12 months after resection^104^.

Although intensive studies have discovered the relationship between miR-135a expression and cancer diagnosis and prognosis, some limitations still exist; the concentration of circulating miRNA is very low, making it difficult to accurately detect in clinical practice, and the expressions of miR-135a in serum and tissues are not always consistent. In addition, the changes in miR-135a expressions are not the same in different cancers. Therefore, further studies are needed before miR-135a can be used as a biomarker in the clinic.

### Perspectives about the potential application of miR-135a in cancer treatment

The tumor suppressive or oncogenic function of miRNAs in cancer indicates that they may serve as therapeutic agents or targets in cancer treatment^105^. As previously discussed, most preclinical studies showed an increased or decreased expression of miR-135a *via* miRNA mimics or inhibitors of miRNA (also known as antagomirs) through systemic or local injection. However, the application of miR-135a in cancer treatment is still faced with challenges. First, the choice of miR-135a mimics or inhibitors is difficult because the expression levels and biological functions of miR-135a vary greatly among different cancers, and changes in the tumor microenvironment also affect miRNA expression^106,107^. To overcome this obstacle, a biopsy (multiple biopsies at different times are better) of the tumor may be necessary to obtain miRNA profiles^3^. Second, the transportation of miR-135a mimics or inhibitors to candidate organs remains a challenge because of their potential degradation by RNases in the serum or endocytic compartment of cells. To address this obstacle, two strategies have been investigated. One is to modify nucleotides or the RNA backbone through chemical modifications, and the other is to develop effective delivery systems, including nanoparticle systems and extracellular vehicles^3,108^. In addition, the targets of miR-135a involve many genes, including oncogenes and tumor suppressor genes. It is difficult to ensure that target oncogenes can be blocked by miRNAs and avoid off-target effects^109,110^. If the systemic use of miRNA mimics or inhibitors changes the expression of genes not involved in cancer, adverse events may occur^111^. Therefore, the targets of miR-135a should be fully investigated before clinical application to ensure the safety of patients.

In addition to miR-135a mimics or inhibitors, miRNA biogenesis enzymes may also be therapeutic targets. Studies have found that dysregulations of miRNA biogenesis enzymes can lead to the abnormal expression of miRNAs and is associated with poor outcomes^112–114^. Restoring these enzymes may inhibit the progression of cancer and improve the prognosis.

It is important to note that although comprehensive preclinical studies have been conducted on miRNAs, few of them have progressed into clinical trials. At present, no clinical trial has explored the application of miR-135a in clinical settings, and the following main problems remain to be solved: identifying the best miRNA targets, designing delivery systems for specific organs, and preventing potential toxicities. However, with the development of genomic engineering and novel delivery platforms, miR-135a therapeutics may become a long-term clinical reality.

## Conclusions

MiR-135a plays dual roles in cancer progression and chemoresistance, and has great potential in cancer diagnosis and treatment. The findings regarding the role of miR-135a in cancer development have been conflicting, and the underlying mechanisms remain to be further explored. Although basic studies have shown that miR-135a is a potential biomarker and therapeutic target for cancer treatment, there are still challenges to be faced in its clinical applications. Therefore, further investigations are required to explore the potential clinical applications of miR-135a, which may facilitate a new era for cancer treatment.

## Figures and Tables

**Table 1:** The miR-135a as a biomarker for cancer diagnosis

Biomarker	Cancer	Sample	Tendency	Accuracy	Reference
MiR-135a	Ovarian cancer	Serum: 47 with ovarian cancer, 14 with ovarian cysts and 7 normal controls	Down-regulated in cancer patients	Not mentioned	^27^
MiR-135a	Colorectal cancer	Serum: 60 with colorectal cancer, 40 with colorectal polyps and 50 normal controls	Up-regulated in cancer patients	AUC^ROC^: 0.832 compared with colorectal polyps; AUC^ROC^: 0.875 compared with normal controls	^21^
MiR-21 MiR-135a MiR-335 MiR-206 Let-7a	Colorectal cancer	Tissues: 15 primary tumor tissues, 19 primary tumor tissues matched with liver metastasis tissues	Up-regulated in liver metastasis	Specificity of 87% and sensitivity of 76% for detection of metastasis	^100^
MiR-200c MiR-605 MiR-135a MiR-433 MiR-106a	Prostate cancer	Serum: 8 with very high-risk prostate cancer, 4 with low-risk one and 4 normal controls	Up: miR-106a, miR-433, miR-200c; Down: miR-605, miR-135a	89% for differentiating very high-risk PC with low-risk and healthy ones	^101^
MiR-135a	Prostate cancer	Tissues: 98 primary prostate cancer tissues, 14 metastatic prostate cancer tissues and 28 normal controls	Down-regulated in metastatic prostate cancer tissues	AUC^ROC^: 0.877 for differentiating metastatic and primary prostate cancer	^29^
Let-7c MiR-148a MiR-204 MiR-135a MiR-135b MiR-345	Bladder cancer	Urine: 115 with bladder cancer and 87 normal controls	Down: let-7c, miR-148a, miR-204; Up: miR-135a, miR-135b, miR-345	AUC^ROC^: 0.883 for differentiating bladder cancer with normal controls	^102^
